# Uropathogen profiles and their antimicrobial resistance patterns in patients: a three-year retrospective study in Sichuan region

**DOI:** 10.3389/fpubh.2025.1493980

**Published:** 2025-02-27

**Authors:** Xiaofang Xu, Yuanfang Wang, Ning Li, Yilei Jin, Xinyi Xu, Zhiwei Zhou, Yi Xie, Qun Sun

**Affiliations:** ^1^Key Laboratory of Bio-Resources and Eco-Environment of the Ministry of Education, College of Life Sciences, Sichuan University, Chengdu, Sichuan, China; ^2^Department of Laboratory Medicine, West China Hospital, Sichuan University, Chengdu, Sichuan, China

**Keywords:** urinary tract infection, urine culture, pathogen, antibiotic, antimicrobial resistance

## Abstract

**Background:**

Urinary tract infection (UTI) is the most common infection requiring empiric antibiotic treatment. Due to the increased antibiotic resistance of uropathogens and their regional variation, monitoring pathogen distribution and antimicrobial susceptibility is important to ensure effective antibiotic therapy. This retrospective study analyzed the 3-year-long uropathogen profiles and their resistance from a single tertiary general hospital (single-center) and 28 hospitals (multi-center) to provide data allowing guidance for appropriate empiric antimicrobial treatment for UTI.

**Methods:**

A total of 26,108 non-repetitive clinical urine isolates from the single-center during 2017–2019 and the multi-center in 2018 were collected, the pathogen and antimicrobial resistance profiles were analyzed.

**Results:**

*Escherichia coli*, *Enterococcus faecium*, *Klebsiella pneumoniae*, *Enterococcus faecalis,* and *Pseudomonas aeruginosa* were the top five bacterial pathogens for both the multi-center and single-center, while the proportion of *Candida albicans* was higher in the single-center. *E. coli* was the most resistant species, with resistance rates exceeding 50% for 13/30 of the antibiotics tested, even exceeding 80% for ampicillin, nalidixic acid and piperacillin. Particularly, the resistance rates of *E. coli* to cefazolin were 62.7% in the multi-center while exceeding 90% in the single-center. Similarly, the resistance rates of *K. pneumoniae* were approximately 40% ~ 60% to 16/29 of the antibiotics tested in the single-center, compared to 30% ~ 50% in the multi-center. In enterococci, *E. faecium* showed the resistance rates exceeding 90% for 6/10 of the antibiotics tested, while *E. faecalis* was highly resistant to erythromycin (> 66%) and tetracycline (> 81%). The main fungal pathogens were *C. albicans*, *Candida tropicalis,* and *Candida glabrata*, with the highest resistance rates exceeding 30% for *C. tropicalis* to fluconazole, itraconazole and voriconazole. The main extended-spectrum beta lactamase (ESBL)-producing isolates were *E. coli* (86.3%) and *K. pneumoniae* (11.3%), with resistance rates exceeding 60% for cephalosporins, sulfonamides, quinolones and tetracycline in the single-center.

**Conclusion:**

*Escherichia coli*, *E. faecium*, *K. pneumoniae*, *E. faecalis*, *P. aeruginosa* and *C. albicans* were the main uropathogens in the southwestern region of China, while *E. coli* and *E. faecium* showed the highest antibiotic resistance. The high resistance of ESBL-producing isolates to cephalosporins, sulfonamides, quinolones and tetracycline in the tertiary general hospital suggests a greater challenge to their antibiotic administration and timely ESBL test, and the empirical antimicrobial therapy should greatly consider the updated local characteristics of the uropathogen resistance, and be more cautious in the tertiary general hospital where patients are more likely to harbor higher resistant pathogens.

## Introduction

Urinary tract infection (UTI), one of the most common infections worldwide and the most common form of bacterial infection (1–3), can occur in the urethra, bladder, ureters or kidneys (2, 4) and be classified as complicated or uncomplicated categories (1, 5). The diagnosis is based on the clinical symptoms, including dysuria, frequency, urgency, pain, pyuria and hematuria (2, 6), and the support of the laboratory tests, such as urinalysis and urine culture (1, 3). Most UTIs are considered easy to treat; however, if not treated promptly or effectively, recurrent episodes cause great physical and mental suffering for patients (7, 8).

Since the traditional urine culture techniques usually require more than 24 h prior to results to be reported and the facility of urine culture is often unavailable in many rural and small-town settings, empiric antibiotics are routinely prescribed (9–13). It has been widely reported that the inappropriate empiric antimicrobial therapy largely contributes to the increased antimicrobial resistance, especially the multidrug-resistant (MDR) and extensively drug-resistant (XDR) pathogens in UTI, which poses a serious threat to the treatment of UTI (4, 13, 14). Because some of the pathogens involved in UTI also cause infection at other sites, resistance may impact the treatment of other infections or other diseases (15).

However, because of the epidemiology, species distribution, and susceptibility patterns varying widely across regions and populations (16–18), antimicrobial management is important for overcoming the problem of resistant pathogens, and the empirical selection of antibiotic therapy for clinicians in UTI requires the knowledge based on local pathogen profiles and up-to-date models of antimicrobial susceptibility (19). Whether the characterization of pathogens isolated from patients in different size hospitals varies, especially among those carried by patients with different disease severity served by different hospitals, has important implications for empirical antibiotic use. Therefore, this study investigating the prevalence and resistance patterns of urinary pathogens over different years and regions in Sichuan (a province with a large population in the southwestern region of China) aimed to provide a reference for the development of better antimicrobial empirical therapies based on similarities and differences and ultimately to improve the treatment of patients with UTI in this region.

## Materials and methods

### Sample collection

Pathogen data from a Grade A tertiary hospital (tertiary general hospital, single-center) from January 2017 to December 2019 and 28 hospitals in Sichuan (multi-center) in 2018 were collected, and the same pathogens cultured from urine samples of the same patients were eliminated. The multi-center data were obtained from the China Antimicrobial Surveillance Network (CHINET). Particularly, the different extended-spectrum beta lactamase (ESBL) results of the same pathogens from the same patients were included. All the urine culture procedures were in accordance with the fourth edition of the National Operating Procedures for Clinical Examination. Since this study was a retrospective analysis of urine data without any detailed personal information, there was no ethics approval.

### Urine culture and antimicrobial susceptibility test

After receiving strain and susceptibility data from the hospitals, the pathogen identification results were checked by the VITEK 2 Compact (BioMérieux, France) and matrix-assisted laser desorption ionization time-of-flight mass spectrometry (MALDI-TOF MS, Bruker Corporation, Germany), and the broth disk method and VITEK 2 Compact were used to check the antimicrobial susceptibility results. The interpretation was based on the Clinical and Laboratory Standards Institute (CLSI) guidelines (20), the standard method of antimicrobial susceptibility and the ESBL test (Supplementary material 1). Quality control was carried out in accordance with CLSI guidelines, and the quality control strains included *E. coli* ATCC25922, *E. coli* ATCC35218, *Staphylococcus aureus* ATCC25923, *Staphylococcus aureus* ATCC29213, *Klebsiella pneumoniae* ATCC700603, *Enterobacter cloacae* ATCC700323, *Pseudomonas aeruginosa* ATCC27853, *Streptococcus pneumoniae* ATCC49619 and *Haemophilus influenzae* ATCC49247. The routine quality control procedure was carried out once a week under stable experimental conditions.

### Statistical analysis

WHONET 5.6 software was used to calculate the isolation rate of the pathogens and their resistance rates to the common antibiotics. GraphPad 8.0 and chi-square test were used for the analysis, and *p* < 0.05 was considered statistically significant.

## Results

### Clinical information of the isolated strains

**Figure 1:**
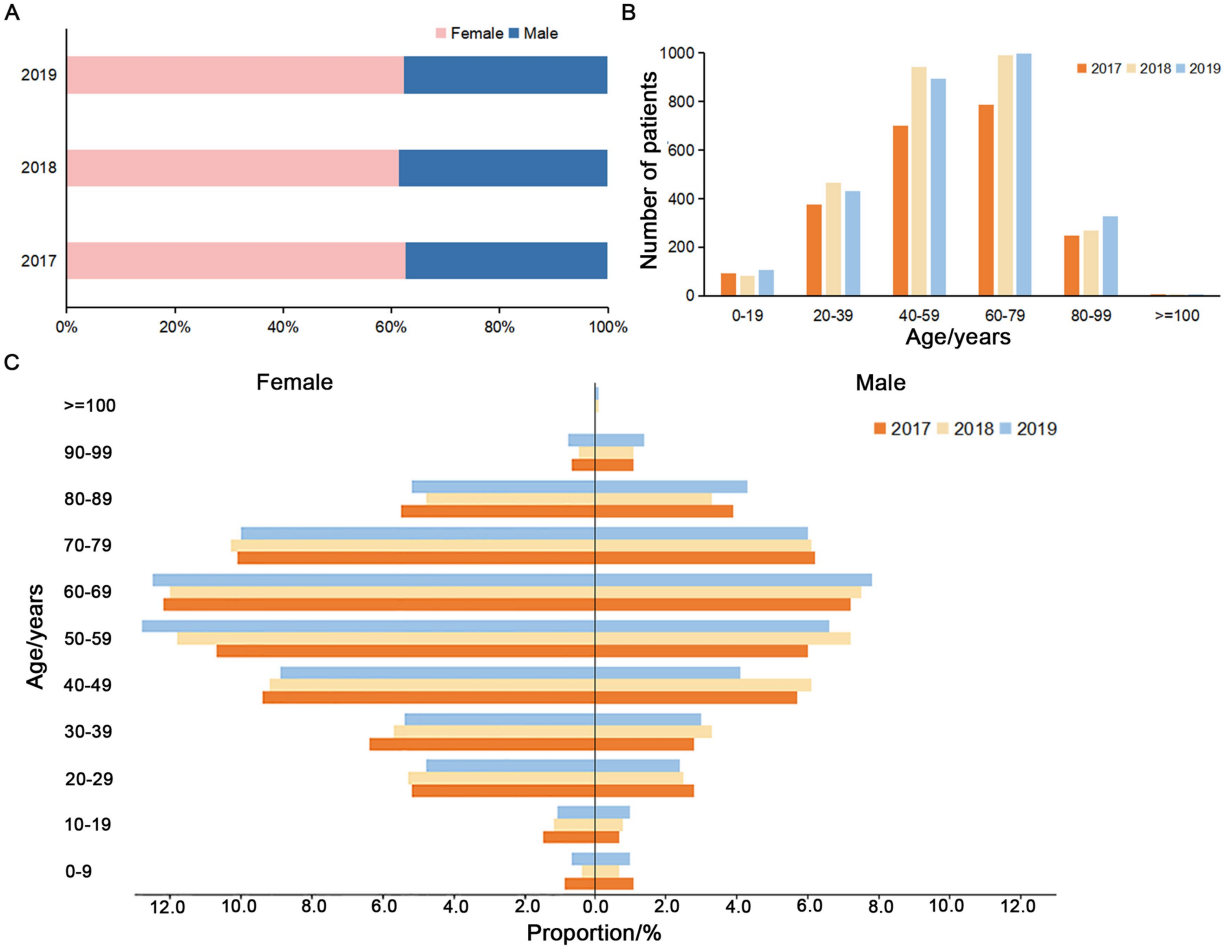
Gender and age composition of the patients from the single-center during 2017–2019. **(A)** Proportion of non-repetitive female and male patients. **(B)** Age composition of the non-repetitive patients. **(C)** Proportion of the non-repetitive male and female patients in different age groups during 2017–2019.

In this study, a total of 9,161 isolates from a tertiary general hospital (single-center, which is usually attended by patients with difficult and complicated diseases due to the advantages in technical strength and equipment conditions of the hospital) during 2017–2019 and 16,952 isolates from 28 hospitals in Sichuan (multi-center) during 2018 were retrospectively analyzed. Among the patients in the single-center from 2017 to 2019, the male/female ratio (0.60,1, 0.63:1 and 0.61:1), age range (0–111, 0–105 and 0–106 years old) and average age (55.61 ± 20.08, 55.93 ± 19.04 and 56.69 ± 19.70 years) for each year were approximately similar (Figures 1A,B). The results showed that the percentage of pathogens detected in females was higher than that in males, and the majority of patients were 40–79 years old (Figures 1B,C). From 20–29 to 60–69 years old, the detection rates of the pathogens increased with age (Figure 1C).

In terms of the detection rates of pathogens from 2017 to 2019, the urology, nephrology, neurology, emergency and infectious disease department all ranked in the top seven (Table 1). The main pathogens were Gram-negative bacteria, and the detection rates exceeded 60% (66.6, 63.6 and 62.1%) (Table 2). From 2017 to 2019, the three main departments in which the pathogens were detected in urine (both the Gram-negative and Gram-positive bacteria detection rates >10%) were the urology, nephrology and emergency department, while the fungi were found in the infectious disease, emergency and respiratory department (Table 2).

**Table 1:** Department distribution of urine samples during 2017–2019 from the single-center.

2017 (*N* = 2,539)		2018 (*N* = 3,283)		2019 (*N* = 3,334)	
Department	Proportion/%	Department	Proportion/%	Department	Proportion/%
Urology	17.4	Urology	14.9	Urology	16.6
Nephrology	12.2	Rehabilitation	10.8	Emergency	14.4
Geriatrics	7.2	Emergency	9.8	Nephrology	9.4
Neurology	7.1	Nephrology	9.6	Neurology	7.2
Emergency	6.9	Neurology	6.4	Geriatrics	6.4
Infectious Disease	6.4	Geriatrics	5.7	Endocrinology	4.7
Endocrinology	3.9	Infectious Disease	5.0	Infectious Disease	4.4
Rheumatology	3.7	Neurosurgery	3.6	Rheumatology	4.2
Outpatient	3.2	Rheumatology	3.1	Respiratory	3.8
Orthopedics	3.2	Endocrinology	3.1	Special Care	3.3
Other	29.0	Other	27.9	Other	25.5

**Table 2:** Distribution of the top seven departments of isolated uropathogens from the single-center during 2017–2019.

	G-proportion/%		G+ proportion/%		Fungi proportion/%	
2017 (*N* = 2,539)	66.6 (*n* = 1,690)		19.7 (*n* = 500)		13.7 (*n* = 348)	
	Urology	19.2	Urology	20	Infectious Disease	17.3
	Nephrology	13.1	Nephrology	13.8	Emergency	15.2
	Neurology	7.5	Neurology	8.2	Respiratory	9.5
	Geriatrics	7.4	Infectious Disease	7.8	ICU	8.3
	Emergency	5.9	Geriatrics	7.6	Nephrology	5.7
	Endocrinology	4.6	Emergency	4.8	Geriatrics	5.5
	Rheumatology	4.2	Respiratory	3.6	Urology	4.9
2018 (*N* = 3,283)	63.6 (*n* = 2087)		19.8 (*n* = 652)		16.6 (*n* = 544)	
	Urology	15.6	Urology	18.9	Emergency	13.9
	Rehabilitation	12.4	Nephrology	12.7	Infectious Disease	10.6
	Nephrology	9.9	Emergency	11	Respiratory	9.7
	Emergency	8.3	Neurology	7.7	Rehabilitation	8.8
	Geriatrics	6	Rehabilitation	7.5	Urology	7.2
	Neurology	5.9	Geriatrics	6.7	Neurosurgery	7
	Rheumatology	4.2	Infectious Disease	4.3	ICU	6.4
2019 (*N* = 3,334)	62.1 (*n* = 2070)		22.7 (*n* = 756)		15.2 (*n* = 508)	
	Urology	19	Urology	17	Emergency	25.4
	Emergency	11.9	Emergency	13.7	Infectious Disease	10.4
	Nephrology	10.4	Nephrology	10.6	Respiratory	8.1
	Neurology	6.9	Geriatrics	8.1	ICU	7.9
	Geriatrics	6.4	Neurology	7.8	Neurology	7.3
	Endocrinology	5.5	Respiratory	6.3	Urology	6.3
	Rheumatology	5.3	Endocrinology	3.7	Neurosurgery	5.7

G−, Gram-negative bacteria; G+, Gram-positive bacteria; ICU, intensive care unit.

### Distribution of the main pathogens

In the single-center during 2017–2019, the detection rates of *Escherichia coli* (38.3% ~ 40.9%), *Enterococcus faecium* (8.5% ~ 10.6%), *Klebsiella pneumoniae* (6.7% ~ 8.3%) and *Candida albicans* (5.0% ~ 5.8%) all ranked in the top four, and *Enterococcus faecalis* (3.7% ~ 4.4%), *Pseudomonas aeruginosa* (3.2% ~ 3.4%), *Candida tropicalis* (2.7% ~ 3.7%) and *Candida glabrata* (1.8% ~ 4.2%) all ranked in the top 10 (Table 3). However, the top 10 pathogens detected in the multi-center in Sichuan in 2018 were *E. coli* (45.3%), *E. faecium* (9.8%), *K. pneumoniae* (7.7%), *E. faecalis* (5.6%), *P. aeruginosa* (2.9%), *Proteus mirabilis* (2.5%), *Enterobacter cloacae* (2.2%), *C. albicans* (2.1%), *Staphylococcus epidermidis* (1.5%), and *Acinetobacter baumannii* (1.4%) (Table 3), which were consistent with eight of the top 10 bacteria, including *E. coli*, *E. faecium*, *K. pneumoniae*, *E. faecalis*, *P. aeruginosa*, *P. mirabilis*, *E. cloacae*, *S. epidermidis*, *Staphylococcus aureus* and *Staphylococcus haemolyticus*, reported by the 83 members of the Bacterial Drug Resistance Monitoring Network in Sichuan in 2018 (21). Compared with the most common uropathogens in the single-center, six of the top 10 were consistent but the detection rate of *C. albicans* was lower in the 28 hospitals in Sichuan in 2018.

**Table 3:** Composition of the top 10 uropathogens in Sichuan during 2017–2019.

Single-center						Multi-center	
2017 (*N* = 2,539)		2018 (*N* = 3,283)		2019 (*N* = 3,334)		2018* (*N* = 16,952)	
Pathogen	Proportion/%	Pathogen	Proportion/%	Pathogen	Proportion/%	Pathogen	Proportion/%
*E. coli*	40.9	*E. coli*	38.5	*E. coli*	38.3	*E. coli*	45.3
*E. faecium*	8.5	*E. faecium*	9.4	*E. faecium*	10.6	*E. faecium*	9.8
*K. pneumoniae*	8.0	*K. pneumoniae*	8.3	*K. pneumoniae*	6.7	*K. pneumoniae*	7.7
*C. albicans*	5.0	*C. albicans*	5.5	*C. albicans*	5.8	*E. faecalis*	5.6
*E. faecalis*	3.7	*C. glabrata*	4.2	*E. faecalis*	4.4	*P. aeruginosa*	2.9
*P. aeruginosa*	3.2	*E. faecalis*	3.8	*C. tropicalis*	3.7	*P. mirabilis*	2.5
*C. tropicalis*	2.7	*P. aeruginosa*	3.4	*C. glabrata*	3.3	*E. cloacae*	2.2
*P. mirabilis*	2.4	*C. tropicalis*	3.4	*P. aeruginosa*	2.8	*C. albicans*	2.1
*C. glabrata*	1.8	*A. baumannii*	1.9	*P. mirabilis*	1.7	*S. epidermidis*	1.5
*A. baumannii*	1.7	*E. cloacae*	1.6	*E. cloacae*	1.4	*A. baumannii*	1.4
Other	22.1	Other	20.2	Other	21.2	Other	19.1

2018*, the data from 28 hospitals in Sichuan in 2018.

*Escherichia coli*, *E. faecium*, *K. pneumoniae*, *C. albicans*, *E. faecalis*, *P. aeruginosa*, *C. tropicalis,* and *C. glabrata* ranked among the top 10 pathogens in the single-center for every year from 2017 to 2019 (Table 3), and their distribution in females and males was shown in Table 4. The pathogen proportions of females to males (Female/Male, F/M) were 1.62 (1,569/970), 1.49 (1965/1318), and 1.59 (2048/1286) from 2017 to 2019, respectively, among which *E. coli*, *E. faecium*, *K. pneumoniae*, *E. faecalis*, *P. aeruginosa*, *C. tropicalis,* and *C. glabrata* showed the similar distribution in females and males. The F/M ratios of *E. coli, E. faecium,* and *C. glabrata* were higher than the total F/M ratios, indicating that these three pathogens infected more females; while the F/M ratios of *K. pneumoniae*, *E. faecalis*, *P. aeruginosa*, and *C. tropicalis* were lower than the total F/M ratios, suggesting that these four pathogens infected more males. In addition, the detection rates of *E. coli*, *E. faecalis,* and *P. aeruginosa* were significantly different between female and male (*p* < 0.05), implying that *E. coli* may prefer to infect the female patients, while *E. faecalis* and *P. aeruginosa* may prefer to infect the male.

**Table 4:** Gender composition of the top 10 isolates during 2017–2019 from the single-center.

Pathogen	2017						2018						2019					
	Female (%)		Male (%)		*p*-value	F/M	Female (%)		Male (%)		*p*-value	F/M	Female (%)		Male (%)		*p*-value	F/M
Total	*n* = 1,569		*n* = 970			1.62	*n* = 1965		*n* = 1,318			1.49	*n* = 2048		*n* = 1,286			1.59
*E. coli*	777	(49.5)	262	(27.0)	<0.0001	2.97	932	(47.4)	331	(25.1)	<0.0001	2.82	987	(48.2)	291	(22.6)	<0.0001	3.39
*E. faecium*	135	(8.6)	81	(8.4)	0.8238	1.67	208	(10.6)	99	(7.5)	0.0030	2.10	226	(11.0)	126	(9.8)	0.2578	1.79
*K. pneumoniae*	117	(7.5)	85	(8.8)	0.2374	1.38	135	(6.9)	136	(10.3)	0.0004	0.99	137	(6.7)	87	(6.8)	0.9323	1.57
*C. albicans*	89	(5.7)	37	(3.8)	0.0362	2.41	107	(5.4)	73	(5.5)	0.9083	1.47	103	(5.0)	89	(6.9)	0.0225	1.16
*E. faecalis*	46	(2.9)	49	(5.1)	0.0062	0.94	55	(2.8)	71	(5.4)	0.0002	0.77	63	(3.1)	85	(6.6)	<0.0001	0.74
*P. aeruginosa*	30	(1.9)	50	(5.2)	< 0.0001	0.60	40	(2.0)	71	(5.4)	<0.0001	0.56	35	(1.7)	57	(4.4)	<0.0001	0.61
*C. tropicalis*	35	(2.2)	34	(3.5)	0.0550	1.03	47	(2.4)	64	(4.9)	0.0001	0.73	63	(3.1)	61	(4.7)	0.0133	1.03
*C. glabrata*	33	(2.1)	13	(1.3)	0.5388	2.54	96	(4.9)	41	(3.1)	0.0127	2.34	86	(4.2)	25	(1.9)	0.0004	3.44

F/M, the proportion of the same pathogen isolated from female and male urine; *p* < 0.05, statistically significant difference.

### Antimicrobial resistance analysis

#### Escherichia coli

A total of 30 antibiotics were counted in the antibiotic susceptibility of *E. coli* (Table 5), among which 13 drugs, including ampicillin, trimethoprim/sulfamethoxazole and ciprofloxacin, etc. showed the resistance rates >50% in the multi-center and single-center; particularly, the resistance rates of ampicillin, nalidixic acid and piperacillin were nearly 80% or more and that of cefazolin was >91%, suggesting that the clinical application of them should be based on the susceptibility results or even suspended (22). Moreover, for gentamicin, aztreonam and ampicillin/sulbactam, which showed resistance rates >30% and <50%, the warning information should be reported or even empirical use should be cautious (22).

**Table 5:** Resistance of *Escherichia coli* and *Klebsiella pneumoniae* to antibiotics.

Antibiotic	*Escherichia coli*/%				*Klebsiella pneumoniae*/%			
	Multi-center	Single-center			Multi-center	Single-center		
	2018*	2017	2018	2019	2018*	2017	2018	2019
Amikacin	2.1	1.7	2.2	1.8	10.7	12.9	17.2	14.4
Gentamicin	38.2	39.6	38.5	35.2	30.2	37.4	37.2	39.8
Tobramycin	13.5	12.6	11.9	11.3	18.2	25.8	24.0	21.3
Ampicillin	86.1	92.5	85.0	86.4	–	–	–	–
Piperacillin	79.4	81.2	81.6	82.7	–	61.0	55.6	62.2
Aztreonam	35.3	34.1	34.9	33.3	34.0	40.7	41.3	40.8
Nitrofurantoin	3.2	2.5	2.8	2.9	40.4	37.4	45.9	44.2
SXT	–	52.8	54.9	51.6	–	47.8	46.3	49.3
Doripenem	0.9	0.9	0.8	1.7	17.5	13.3	19.5	17.5
Ertapenem	1.1	1.1	1.3	1.5	7.3	12.6	19.8	16.7
Imipenem	2.1	0.7	0.9	1.5	10.1	13.5	18.7	16.2
Meropenem	1.1	0.8	1.0	1.4	15.3	13.6	19.4	17.1
Minocycline	16.5	26.6	18.1	17.9	26.2	36.4	28.7	54.6
Tetracycline	62.9	61	59.9	61.6	49.3	56.8	46.2	57.9
TZP	3.3	5.8	6.3	5.1	13.5	24.7	31.4	26.2
SAM	43.2	50.3	48.8	49.2	–	57.5	53.6	58.8
AMC	9.7	9.7	10.7	10.5	22.2	25.2	34.0	30.4
TCC	19.2	18.8	17.4	13.7	–	35.6	36.5	35.3
Cefepime	24.2	15.7	15.2	12.2	26.6	24.1	29.6	27.3
Cefpodoxime	55.4	55.8	55.3	54.3	–	56.8	50.8	53.9
Cefuroxime	55.9	56.2	56	54.7	–	57.2	53.3	55.8
Ceftriaxone	52.7	54.4	54.4	53.8	44.7	53.1	48.2	50.7
Cefotaxime	56.5	55.2	54.5	53.7	–	53.1	50.3	52.5
Ceftazidime	24.4	25.0	22.6	29.4	28.9	31.3	35.9	33.6
Cefotetan	2.4	1.9	2.8	2.0	–	11.0	19.4	17.9
Cefazolin	62.7	91.3	94.3	91.1	–	94.9	97.2	96.8
Nalidixic acid	88.0	85.5	87.9	87.9	–	49.3	48.5	49.3
Levofloxacin	51.9	59.1	59.0	60.9	30.9	43.8	39.3	42.1
Norfloxacin	56.9	59.2	59.6	61.3	41.2	44.8	38.8	43.9
Ciprofloxacin	54.9	61.2	61.4	62.9	34.4	47.2	41.0	46.1

2018*, the data from 28 hospitals in Sichuan in 2018; “–” represents no result of the antibiotic. SXT, trimethoprim/sulfamethoxazole; TZP, piperacillin/tazobactam; SAM, ampicillin/sulbactam; AMC, amoxicillin/clavulanate; TCC, ticarcillin/clavulanate.

#### Klebsiella pneumoniae

A total of 29 antibiotics were counted in the antibiotic susceptibility of *K. pneumoniae* (Table 5), among which 10 drugs, including gentamicin, aztreonam, nitrofurantoin, ceftazidime, nalidixic acid, trimethoprim/sulfamethoxazole, ticarcillin/clavulanate, levofloxacin, norfloxacin and ciprofloxacin, showed the resistance rates >30% but <50%, suggesting that warning information should be reported or even empirical use should be cautious (22). Regarding the drugs with resistance rates >50% but <75%, piperacillin, tetracycline, cefpodoxime, cefuroxime, cefotaxime, ceftriaxone and ampicillin/sulbactam in the multi-center and single-center suggested that clinical application should be based on the susceptibility results (22). In particular, the resistance rates of cefazolin in the single-center during 2017–2019 were >94% (94.9, 97.2 and 96.8%), suggesting that its clinical application should be suspended (22).

#### Pseudomonas aeruginosa

A total of 12 antibiotics were counted in the antibiotic susceptibility of *P. aeruginosa* in the multi-center and single-center (Table 6), among which the resistance rates of the most antibiotics were <30% except that ticarcillin/clavulanate with the resistance rates >30% but <50% was reported in the single-center in 2017 and the multi-center, suggesting that warning information should be reported or even empirical use should be cautious (22).

**Table 6:** Resistance of *Pseudomonas aeruginosa* to antibiotics.

Antibiotic	Multi-center/%	Single-center/%		
	2018*	2017	2018	2019
Amikacin	2.3	12.7	5.1	5.9
Gentamicin	9.4	17.5	12.8	7.1
Tobramycin	-	15.0	14.3	7.2
Piperacillin	20.5	25.5	14.5	17.1
Aztreonam	21.7	29.3	14.1	15.5
Meropenem	11.3	19.4	10.3	12.8
Imipenem	10.8	20.6	12.8	12.9
Ticarcillin/Clavulanate	33.6	38.0	26.0	22.6
Piperacillin/Tazobactam	6.7	22.2	13.2	11.1
Ciprofloxacin	16.5	21.9	19.2	16.7
Levofloxacin	12.6	23.0	19.5	15.5
Norfloxacin	13.1	17.6	15.3	15.0

2018*, the data from 28 hospitals in Sichuan in 2018; “–” represents no result of the antibiotic.

#### Enterococcus faecium

A total of 10 antibiotics were counted in the antibiotic susceptibility of *E. faecium* in the multi-center and single-center (Table 7), among which the resistance rates to erythromycin, ciprofloxacin, moxifloxacin, levofloxacin, ampicillin and penicillin (60%, 6/10) in the multi-center and single-center were nearly 90% or more, suggesting that clinical application should be suspended (22). Tetracycline and nitrofurantoin with the resistance rates of approximately 50%, suggested that the clinical application should be based on the susceptibility results or the empirical use should be cautious (22). Relatively fortunately, the resistance rates to linezolid and vancomycin were <4%. Interestingly, according to the susceptibility results in the single-center, *E. faecium* was 100% resistant to ampicillin in 2017 and 100% resistant to erythromycin in 2019.

**Table 7:** Resistance of *Enterococcus* to antibiotics.

Antibiotic	*Enterococcus faecium*/%				*Enterococcus faecalis*/%			
	Multi-center	Single-center			Multi-center	Single-center		
	2018*	2017	2018	2019	2018*	2017	2018	2019
Erythromycin	–	90.1	90.7	100.0	76.6	76.3	66.7	–
Linezolid	0.2	–	0.4	0.3	1.6	6.6	2.9	8.3
Ciprofloxacin	93.2	96.3	97.8	97.4	33	25.3	24	30.1
Moxifloxacin	96.5	–	–	–	–	–	–	–
Levofloxacin	92.3	95.7	97.4	96.5	26.8	24	23.1	29.3
Ampicillin	94.7	100.0	98.1	97.5	–	–	1.0	0.0
Penicillin	–	96.9	98.9	98.4	–	5.3	3.9	3.0
Tetracycline	50.2	46.3	48.1	44.7	81.1	90.7	85.4	83.5
Vancomycin	1.6	3.2	3.0	2.6	0.6	1.4	0.0	0.0
Nitrofurantoin	48.5	48.4	51.3	51.8	4.3	1.4	1.9	1.5

2018*, the data from 28 hospitals in Sichuan in 2018; “–” represents no result of the antibiotic.

#### Enterococcus faecalis

A total of nine antibiotics were counted in the antibiotic susceptibility of *E. faecalis* in the multi-center and single-center (Table 7). The resistance rates to the antibiotics except erythromycin, ciprofloxacin, levofloxacin and tetracycline were <20%, among which the resistance rate to tetracycline was >80%, erythromycin was nearly 70% and ciprofloxacin was nearly 30% or more, suggesting that the clinical application of tetracycline and erythromycin should be suspended and ciprofloxacin should be warned (22). Specifically, *E. faecalis* in the single-center was 100% sensitive to ampicillin in 2019 and vancomycin in 2018 and 2019.

### Main fungi

The main pathogenic fungi isolated from the urine in the single-center during 2017–2019 were *C. albicans*, *C. tropicalis,* and *C. glabrata*. The three main pathogenic fungi were 100% sensitive to 5-flucytosine (Table 8). The comparison of the resistance rates of the three pathogens to the antifungals showed that the resistance rates of *C. albicans* were <7%, those of *C. glabrata* were 2.2% ~ 17.0%, and those of *C. tropicalis* to the antifungals except amphotericin B were >30%.

**Table 8:** Resistance of the main fungi to antifungals.

Pathogen	Antifungal	2017		2018		2019	
		Strains*	R/%	Strains*	R/%	Strains*	R/%
*C. albicans*	FLC	37	2.7	72	6.9	94	3.2
	ITC	37	2.7	72	6.9	94	3.2
	VRC	37	2.7	72	6.9	94	3.2
	AMB	37	0.0	72	0.0	94	1.1
	5FC	37	0.0	–	–	94	0.0
*C. tropicalis*	FLC	26	38.5	53	41.5	83	47.0
	ITC	26	30.8	53	35.8	83	31.3
	VRC	26	34.6	53	35.8	83	47.0
	AMB	26	0.0	53	0.0	83	1.2
	5FC	26	0.0	–	–	83	0.0
*C. glabrata*	FLC	20	15.0	44	6.8	53	11.3
	ITC	20	15.0	45	8.9	53	17.0
	VRC	20	5.0	45	4.4	–	–
	AMB	20	0.0	45	0.0	53	7.5
	CAS	17	0.0	45	2.2	–	–
	5FC	20	0.0	–	–	53	0.0

Strains*, the total number of the isolates involved in the antimicrobial susceptibility test. R, resistance rate. “–” represents no result of the antifungal. AMB, amphotericin B; CAS, caspofungin; 5FC, 5-flucytosine; FLC, fluconazole; ITC, itraconazole; VRC, voriconazole.

### Extended-spectrum beta lactamase detection

Analysis of extended-spectrum beta lactamase (ESBL)-producing strains in the single-center from 2017 to 2019 was shown in Table 9. The positive rates of ESBL from 2017 to 2019 were 49.8% (499/1002), 49.9% (634/1271) and 49.7% (707/1422), respectively. The main ESBL-producing strains were *E. coli* (86.3%, 1587/1840), *K. pneumoniae* (11.3%, 208/1840), *P. mirabilis* (1.5%, 27/1840) and *Klebsiella oxytoca* (0.9%, 16/1840) during 2017–2019 (Table 9). According to the ESBL tests during 2017–2019, the proportion of ESBL+ in *E. coli* (ESBL-ECO) accounted for 51.7% (51.5, 52.3 and 51.2%, respectively), while the proportion of ESBL+ in *K. pneumoniae* (ESBL-KPN) accounted for 43.3% (48.6, 38.4 and 42.9%, respectively). ESBLs-producing *P. mirabilis* and ESBL-producing *K. oxytoca* accounted for 38.3% (22.6% ~ 52.4%) and 26.4% (21.1% ~ 31.3%), respectively. The ESBL-ECO and ESBL-KPN were highly resistant to cefpodoxime, ceftriaxone, cefotaxime, cefuroxime, cefazolin and piperacillin, with resistance rates exceeding 91% (Table 10). In addition, the two had the resistance rates exceeding 50% to a variety of antibiotics, including nalidixic acid, ampicillin/sulbactam, tetracycline, ciprofloxacin, levofloxacin, norfloxacin, aztreonam and trimethoprim/sulfamethoxazole; particularly, ESBL-ECO were strongly resistant to nalidixic acid with a resistance rate > 92% (Table 10).

**Table 9:** Distribution of the ESBL-producing isolates and ESBL alteration in the same patient from the single-center.

Pathogen	2017 (*N* = 1,002)		2018 (*N* = 1,271)		2019 (*N* = 1,422)		2017–2019		ESBL alteration		
	ESBL+/%		ESBL+/%		ESBL+/%		Total *	Proportion/%*	ESBL + to −	ESBL − to +	Two alterations
ECO	418	51.5	559	52.3	610	51.2	1,587	86.3	22	30	5
KPN	69	48.6	61	38.4	78	42.9	208	11.3	2	3	1
PMI	7	22.6	9	40.9	11	52.4	27	1.5	0	0	0
KOX	5	31.3	4	21.1	7	26.9	16	0.9	1	0	0
Other	0	0.0	1	50.0	1	50.0	2	0.1	0	0	0
Total*	499		634		707		1840		25	33	6

ECO, *E. coli*; KPN, *K. pneumoniae*; PMI, *P. mirabilis*; KOX, *K. oxytoca*. Total*, the total ESBL+ isolates for 2017, 2018, 2019 or the total ESBL+ isolates of the specific species during 2017–2019. Proportion/%*, the proportion of the specific ESBL+ isolates in the total ESBL+ isolates during 2017–2019. ESBL + (−) to – (+), the changes of two repetitive isolates from the same patient from ESBL+ (−) to ESBL− (+) during the same year; two alterations, the changes of three repetitive isolates from the same patient from ESBL− (+) to ESBL+ (−), and then to ESBL− (+) during the same year.

**Table 10:** Resistance of ESBL-ECO and ESBL-KPN to antibiotics.

Antibiotic	ESBL-ECO						ESBL-KPN					
	2017		2018		2019		2017		2018		2019	
	Strains*	R/%	Strains*	R/%	Strains*	R/%	Strains*	R/%	Strains*	R/%	Strains*	R/%
Gentamicin	194	46.9	253	46.3	229	38.9	40	58.8	28	46.7	47	61.8
Ampicillin	–	–	541	99.4	586	99.8	–	–	–	–	–	–
Piperacillin	344	99.7	540	99.3	584	99.2	62	98.4	60	100.0	75	97.4
Aztreonam	249	60.4	340	62.3	357	60.7	47	69.1	42	70.0	50	64.9
Trimethoprim/Sulfamethoxazole	265	64.0	352	65.1	355	60.5	54	79.4	44	75.9	60	77.9
Minocycline	96	30.5	107	20.7	378	70.1	32	51.6	27	52.9	27	37.0
Tetracycline	234	67.8	363	66.6	397	67.4	55	87.3	46	76.7	64	84.2
Ampicillin/Sulbactam	226	65.1	348	63.7	346	58.8	54	85.7	51	86.4	69	89.6
Ticarcillin/Clavulanate	81	23.4	120	22.0	79	13.4	28	44.4	26	43.3	29	38.7
Ceftazidime	129	41.3	201	36.8	303	51.5	26	47.3	27	46.6	33	42.9
Cefpodoxime	343	99.4	530	97.2	580	98.5	61	96.8	55	91.7	73	94.8
Ceftriaxone	408	98.6	534	98.2	582	99.0	65	95.6	55	93.2	71	92.2
Cefotaxime	343	99.4	533	97.8	438	99.5	61	96.8	56	93.3	62	91.2
Cefuroxime	343	99.4	537	98.4	586	99.7	62	98.4	56	93.3	71	93.4
Cefazolin	404	99.0	542	99.8	580	100.0	67	100.0	58	98.3	73	100.0
Cefepime	107	25.8	130	23.8	105	17.9	21	30.9	19	32.2	21	27.6
Nalidixic acid	321	92.8	514	94.3	557	94.7	42	66.7	35	58.3	53	69.7
Levofloxacin	315	76.1	400	73.5	445	75.6	42	61.8	30	50.8	45	58.4
Norfloxacin	260	75.4	402	73.8	379	75.0	39	62.9	30	50.8	44	67.7
Ciprofloxacin	323	78.0	413	75.8	456	77.4	46	67.6	32	54.2	52	67.5

ESBL-ECO, ESBLs-producing *E. coli*; ESBL-KPN, ESBLs-producing *K. pneumoniae*; Strains*, the number of the resistant ESBL-strains; R, resistance rate.

According to the ESBL results from the single-center during the same year from 2017 to 2019, the urine samples from 52 patients showed inconsistent ESBL results for the same pathogen, among which 19 patients showed the first ESBL+ and second ESBL−, 27 patients showed the first ESBL− and second ESBL+, and six patients presented with three ESBL results alternating between the positivity and negativity in the same year (Table 9), which demonstrated that resistance might have changed within 1 year in the patients despite infection with the same pathogen.

## Discussion

This study demonstrated the distribution of the main pathogens and their resistance in urine samples from a single tertiary general hospital and multiple hospitals in Sichuan, China. The key to antibiotic therapy is timely and proper antibiotic administration, but the fact that waiting for delayed susceptibility results requires the empirical use of antibiotics (1) may present inaccuracies. Moreover, a study showed more frequent isolation of the resistant pathogens after antibiotic exposure (23), demonstrating the importance of empirical antibiotic accuracy. Additionally, the notification of the Health and Family Planning Commission of the People’s Republic of China recommended the use of antibiotics by grading the resistance rate to further strengthen the management of antibiotic clinical application (22). While the geographical distribution and resistance of uropathogens varied (4, 16), it is quite important to investigate the local prevalence and antibiotic resistance of pathogens. According to the comparison of data between the single-center and multi-center, the similarities and differences between the two can provide a reference for a more effective empirical use of antibiotics for treating UTI.

Similar to the previous studies (18, 21), more positive samples were found in females than in males, which is also consistent with the fact that UTIs occur more frequently in females (4, 24). In addition, positive samples were mostly obtained from middle-aged and older adults, which is consistent with the findings of Huang et al. (4). Moreover, a previous study (22) proposed that the distributions of pathogenic bacteria in different departments of hospital were markedly different. According to the results of this study, the positive urine samples were collected mainly from the urology and nephrology department, while the fungal samples were collected mainly from the infectious disease, emergency and respiratory department. The results suggested that different departments should focus on the prevention and control of different species of pathogens.

The top four pathogens in the single-center remained unchanged from 2017 to 2019, and its top five bacteria were consistent with the 28 hospitals in Sichuan in 2018 (Table 3), also consistent with the other multi-center in Sichuan in 2018 (21, 25) and during 2011–2012 (26), China Antimicrobial Resistance Surveillance System (CARSS) (18), and other cities (4, 22, 27), but a little different from several studies (28–31). Due to the existence of bacterial and fungal resistance surveillance networks, most previous studies in China have focused only on the distribution of bacteria in urine, while a few studies mentioned the distribution of fungi (27, 30), among which *C. albicans* was the most common fungus in urine, and also the main pathogen of candidemia and invasive candidiasis (32, 33). Compared with the single-center, the percentage and diversity of fungi in the multi-center in Sichuan were lower (Table 3). Since the single-center is one of the preeminent hospitals in China, mainly serving patients with complex and challenging conditions, the fungal distribution in the single-center may be related to the severe disease and wide source range of patients.

It is well known that UTIs occur more frequently in females than in males, and most studies focused on the gender distribution of UTIs and paid less attention to the distribution of different pathogens in males and females. This study showed that the proportions of *E. coli*, *E. faecium,* and *C. glabrata* were higher in females, while the proportions of *K. pneumoniae*, *E. faecalis*, *P. aeruginosa,* and *C. tropicalis* were higher in males in the single-center from 2017 to 2019, the previous studies also showed similar trends (4, 21). In addition, CARSS (18) reported the composition of urinary pathogenic bacteria in China, among which *E. coli* was the top one species in both males and females, but the number of isolates from females was higher than that from males; moreover, the detection rates of *E. faecalis* and *P. aeruginosa* were top two and five in males but top four and six in females. The UTI-related studies showed similar distributions of pathogens (34, 35). Furthermore, this study found significant differences in the detection rates of *E. coli*, *E. faecalis,* and *P. aeruginosa* between the males and females in 3 years (*p* < 0.05), indicating that the gender may lead to different distribution of pathogens.

This study showed that the resistance of *E. coli* among the main Gram-negative bacteria in Sichuan was strong, with the resistance rates to ampicillin, nalidixic acid, piperacillin and cefazolin reaching 80%. The reported resistance rates of urinary *E. coli* to ampicillin were also nearly 80% or higher (4, 18, 21, 25, 29–31, 36, 37), while some >90% (29, 30) and a few <71% (27, 38). In addition, cefuroxime, ceftriaxone, cefotaxime, levofloxacin and ciprofloxacin with resistance rates of approximately 50% ~ 60% in this study were similar to the results of several studies (4, 18, 24, 27, 29, 37), while Mohamed et al. (39) showed that the resistance rates to trimethoprim/sulfamethoxazole, ceftriaxone and cefuroxime were as high as 80%. Particularly, the resistance rates of *E. coli* to cefazolin in the multi-center were 62.7%, whereas the resistance rates were >90% (91.3, 94.3, and 91.1%) during 2017–2019 in the single-center (Grade A tertiary hospital, Chinese hospital classification) (Table 5). Compared with the previously reported resistance rates of *E. coli* to cefazolin, Feng et al. (21) reported approximately 50% in 86 hospitals in Sichuan Province in 2018, Xia et al. (37) reported 70.5% in three hospitals in Heilongjiang Province, Wu and Zhao (29) reported 90.67% in one Grade A tertiary hospital in Hebei Province, Yang and Lin (30) reported 60.1% in one Grade B tertiary hospital in Sichuan Province, and Liu et al. (38) reported 29% in one secondary hospital in Beijing. Similarly, in bloodstream infections, *E. coli* isolates from tertiary hospitals had higher resistance rates to piperacillin, cefazolin, ciprofloxacin and levofloxacin than those from Sichuan Province bacterial drug resistance monitoring network, the multi-center (40–42). It is hypothesized that the resistance rates vary by hospital grade and number of hospitals, and the higher resistance rates in tertiary hospitals suggest that Grade A tertiary hospitals should be more cautious in terms of the antibiotic use.

The resistance of *K. pneumoniae* was inferior to *E. coli*, as the resistance rates to the most antibiotics were nearly 40% or higher; in particular, the resistance rate to cefazolin in the single-center was >94%, and that to tetracycline in the multi-center was nearly 50%. *K. pneumoniae* showed the resistance rates of approximately 40% ~ 60% for 16/29 of the antibiotics tested in the single-center (tertiary hospital), which were similar to the tertiary hospitals in Beijing (4) and Harbin (37), and had similar trends but lower resistance rates in the multi-center and two other multi-center (21, 25), suggesting that the resistance rates of *K. pneumoniae* in the tertiary hospital may be higher than the total resistance rates in multiple hospitals.

Since the resistance rates of *P. aeruginosa* to almost all antibiotics were < 30%, its resistance was better than *E. coli* and *K. pneumoniae*. Although the resistance rates in the single-center in 2017 were higher than those in 2018 and 2019 (Table 6), the resistance condition was similar to the multi-center in Sichuan. This study was similar to the findings except that the resistance rate to piperacillin/tazobactam was higher than that reported by CARSS (18) and that to aztreonam was lower than that reported by Huang et al. (4). However, several studies reported that the resistance of *P. aeruginosa* was higher, with the resistance rates >30% in Xuzhou (31), >50% to multiple antibiotics in Somalia (39), and >50% to more than half of the antibiotics in Harbin (37). The present study and previous reports seem to demonstrate the variable resistance rates of *P. aeruginosa*.

In the present study, *E. faecium* had a broader spectrum of resistance than *E. faecalis*. *E. faecalis* had high resistance rates to only tetracycline (>81%) and erythromycin (>66%) (Table 7); however, the resistance rates of *E. faecium* to multiple antibiotics were >40% and some of the antibiotics tested were >90% (Table 7). Several studies showed similar trends (4, 24, 27, 29, 30, 37), but two of them were more severe, among which one was the resistance rates of *E. faecium* to the most antibiotics were >63% and the resistance rates of *E. faecalis* to tetracycline, ciprofloxacin and levofloxacin were >51% (37), and the other was the resistance rates of *E. faecium* to multiple drugs were >90% and the resistance rates of *E. faecalis* to ciprofloxacin, levofloxacin and erythromycin were >60% (24). Therefore, antibiotics should be selected on the basis of specific resistance in the treatment of enterococci. The resistance rates of *E. faecium* to ampicillin (*n =* 6) in 2017 and erythromycin (*n =* 2) in 2019 were 100%, which were higher than other studies about *E. faecium*. For example, Wu and Zhao (29) reported the resistance rates to ampicillin and erythromycin were 96.67% (*n =* 174) and 91.67% (*n =* 165); CARSS (18) reported the resistance rates to ampicillin were 93.1% ~ 94.5% (*n* > 19,440); Feng et al. (21) reported the resistance rates to ampicillin were 93.3% ~ 93.4% (*n* > 1,450); and Long et al. (25) reported the resistance rates to ampicillin and erythromycin were 94.7% (*n =* 7,987) and 90.9% (*n =* 7,541). This study with 100% resistance rates may be related to the fact that *E. faecium* is already extremely resistant to ampicillin and erythromycin but the sample size was very small, indicating that a larger sample size should be considered in the future to improve the accuracy of the resistance rates.

In this study (from 2017 to 2019), there were similarities and differences in the antibiotic resistance of five main pathogenic bacteria compared with that reported by 54 hospitals in the Bacterial Resistance Monitoring Network in Sichuan during 2011–2012 (26). For Gram-negative bacteria, ampicillin, piperacillin, trimethoprim/sulfamethoxazole, ampicillin/sulbactam, ticarcillin/clavulanate, tetracycline, ceftriaxone and cefotaxime were still severely resistant to both *E. coli* and *K. pneumoniae* (Resistance rates >43%), while *E. coli* remained severely resistant to levofloxacin (Resistance rates >50%). However, the resistance rates of *E. coli* and *K. pneumoniae* to trimethoprim/sulfamethoxazole (51.6% ~ 54.9% vs. 66.3%, 46.3% ~ 49.3% vs. 53.9%), tetracycline (59.9% ~ 62.9% vs. 71.3%, 46.3% ~ 49.3% vs. 53.9%), ticarcillin/clavulanate (13.7% ~ 19.2% vs. 66.6%, 46.2% ~ 57.9% vs. 64.8%), and cefotaxime (53.7% ~ 56.5% vs. 67.6%, 50.3% ~ 53.1% vs. 69.8%) decreased. For Gram-positive bacteria, both *E. faecium* and *E. faecalis* were still highly resistant to erythromycin and tetracycline (Resistance rates >44%), while *E. faecium* remained highly resistant to ampicillin, ciprofloxacin, moxifloxacin, levofloxacin and nitrofurantoin (Resistance rates >50%). A comparison of resistance rates during 2011–2012 suggests the importance of antibiotic management, and although some antibiotic resistance rates have declined, there are still antibiotics that are severely resistant and strict management of antibiotic use needs to be continued.

In this study (2017–2019), several antibiotics showed high resistance, including ampicillin to *E. coli*, *K. pneumoniae*, and *E. faecalis* (Resistance rates >85%), cefazolin (first-generation cephalosporin) to *E. coli* and *K. pneumoniae* (Resistance rates 62% ~ 93%), and erythromycin, ciprofloxacin, levofloxacin, penicillin and tetracycline to enterococci (Resistance rates >90%). Ampicillin, cefazolin, penicillin, and ofloxacin had been counted as one of the main antimicrobial drugs used in several hospitals during 1997–1999 (43–45), and erythromycin was also listed as one of the main antimicrobial drugs used (43, 44). In addition, Jiang (46) reported that cefazolin, erythromycin, tetracycline, penicillin and levofloxacin were the main antimicrobial drugs used in outpatient clinics in 2000, and Yin and Wen (47) reported that the commonly chosen drugs for uncomplicated UTI included oral dosage forms of fluoroquinolones such as ciprofloxacin and ofloxacin in 2001. It is hypothesized that antibiotics once used for a long period of time may be one of the selection pressures for pathogen resistance. Since antimicrobial drugs have been applied in human and veterinary animals, some veterinary antibiotics that were used for both before but now only for human might bring higher selection pressure for resistant strains, leading to the increase of human antibiotic resistance rate (48). Investigation on the use of veterinary antimicrobial drugs in China during 2010–2017 showed (49) that the total amount of antibiotic use increased year by year, and there was incorrect use of antibiotics; a study of global veterinary antimicrobial drug use showed (50) that the use of veterinary antimicrobial drugs in Asia and the Pacific was almost three times that of Europe, and the most used globally were tetracyclines. These data showed the danger of incorrect use of veterinary antimicrobial drugs to clinical antimicrobial resistance in human. In the face of the increasingly serious antimicrobial resistance in China, the drafting instruction for the Guidelines for the Clinical Application of Antimicrobial Drugs was issued in 2004 (51); in 2005, the banning of the veterinary use of vancomycin, cefoperazone and cefotaxime was issued (48), and the bacterial resistance monitoring network was established (52); the establishment of the National Veterinary Drug Resistance Monitoring Laboratory was initiated in 2002, and the National Animal Bacterial Resistance Monitoring Network was launched in 2008 (53); in 2013, the national standard for food safety, Determination of Erythromycin Residues in Aquatic Products (GB 29684–2013) was released (54); in 2015, the national comprehensive governance of veterinary antimicrobials was initiated (55); and the pilot project of reduction of veterinary antimicrobials use was started in 2018 (56).

Regarding the fungi, the resistance of *C. tropicalis* to fluconazole, itraconazole and voriconazole was higher than that of *C. albicans*. Similar to several studies (29, 33, 57), *C. albicans*, *C. tropicalis* and *C. glabrata* showed 100% sensitivity to 5-flucytosine. However, it was possible that the small number of isolates led to a large deviation in the resistance rates among different studies.

For the multidrug resistance in the single-center from 2017 to 2019, the proportion of ESBL-ECO in the ESBL detection was relatively stable (51.5, 52.3, and 51.2%, respectively), while the proportion of ESBL-KPN fluctuated (48.6, 38.4, and 42.9%, respectively) and that of ESBL-PMI increased annually (22.6, 40.9, and 52.4%, respectively). The positive detection rates of ESBL in the single-center for 3 years were similar to the previous findings (27, 58–60), for example, Cai et al. (59) reported higher ESBL-KPN (63.6%), while Liang and Wang (58) reported a lower ESBL positive rate (30%). ESBL-producing strains were associated with the resistance to cephalosporins, quinolones, tetracyclines and sulfonamides (58–60). In this study, the ESBL-producing strains were highly resistant to ampicillin, aztreonam, ciprofloxacin, cefpodoxime, ceftriaxone, cefotaxime, cefuroxime, cefazolin, levofloxacin, nalidixic acid, norfloxacin, piperacillin, ampicillin/sulbactam, trimethoprim/sulfamethoxazole and tetracycline with the resistance rates >60% and even >99%. Zilberberg et al. (5) proposed that it is useful to know the resistance to one drug might be a marker of resistance to another drug for the same pathogen. Therefore, due to the correlation between the ESBL positivity and high resistance rates to multiple antibiotics, focusing on ESBL strains is significantly valuable for resistance control. In addition, the exclusion criteria for assessing the prevalence of ESBL were not based on the routine elimination method of uropathogens, but on the ESBL characteristics of pathogens, suggesting that the elimination of the same pathogen from the same patient may result in the elimination of the same species but different drug resistance.

This study also had several limitations. The retrospective study in the laboratory prevented further refinement of more detailed information about the patients, and also the multi-center data could not be more detailed. Moreover, only one single center may be biased, and more regional similarities and differences may be found in multiple single-center studies. Furthermore, the multi-center data were available for only 2018, preventing the single-center and multi-center annual comparisons. However, comparative analysis revealing the similarities and differences between the single and multiple is important for understanding the consistency trend and focusing on the particularity of the single center for the formulation of the local management measures. From the One-Health perspective, our findings have important clinical significance, suggesting that the previous non-standard use of antibiotics may contribute the development of the drug resistance, and then the patients with higher resistant pathogens may gather in the tertiary hospitals due to their difficult and complicated symptoms, resulting in the higher drug resistance rates in tertiary hospitals. Therefore, the findings of this study suggest the importance of standardized use of antibiotics in all cases and the need for antibiotic susceptibility testing especially in tertiary hospitals. Accordingly, the commonalities between single-center and multi-center comparative analysis can further provide a reference for the establishment of general and efficient methods, while the particularities also trigger thinking about the applicability of general methods in special circumstances, which can inspire the development of more suitable and efficient schemes. An accurate understanding of the prevalence and resistance of uropathogens is conducive to clinical empirical drug use and may even provide a reference for point-of-care testing. Therefore, regular updates on the distribution and resistance of pathogens are necessary for the treatment of UTI.

## Conclusion

This study demonstrated the uropathogens and their resistance profiles in the single-center and multi-center in the southwestern region of China. *E. coli*, *E. faecium*, *K. pneumoniae*, *E. faecalis,* and *P. aeruginosa* were the top five bacteria, and *C. albicans* was the predominant fungal pathogen. Among the main Gram-negative bacteria, *E. coli* was the most resistant; among the main Gram-positive bacteria, *E. faecium* was more resistant than *E. faecalis*. The resistance of *C. tropicalis* was higher, although the main fungi were *C. albicans*. In the tertiary general hospital, ESBL-producing strains were highly resistant to cephalosporins, quinolones, sulfonamides and tetracycline. The severe resistance of the main pathogens suggests that clinically appropriate empirical antibiotic use needs to be combined with the antibiotic resistance profiles of the local update main pathogens, and even cross-resistance should be considered, which may contribute to the better empirical treatment selection.

## Data Availability

The original contributions presented in the study are included in the article/Supplementary material, further inquiries can be directed to the corresponding authors.
