# Supplementary material for: Uropathogen profiles and their antimicrobial resistance patterns in patients: a three-year retrospective study in Sichuan region
Source: Front Public Health. 2025 Feb 27;13:1493980. doi: 10.3389/fpubh.2025.1493980 (PMC11905819; doi:10.3389/fpubh.2025.1493980)
Supplement: Supplementary file 1 [file Data_Sheet_1.pdf]

CLINICAL AND LABORATORY STANDARDS INSTITUTE®

28th Edition

M100

Performance Standards for Antimicrobial Susceptibility Testing

This document includes updated tables for the Clinical and Laboratory Standards Institute antimicrobial susceptibility testing standards M02, M07, and M11.

A CLSI supplement for global application.

Table 3A. Tests for Extended-Spectrum β-Lactamases in *Klebsiella pneumoniae*, *Klebsiella oxytoca*, *Escherichia coli,* and *Proteus mirabilis*

NOTE: Following evaluation of PK-PD properties, limited clinical data, and MIC distributions, revised breakpoints for cefazolin, cefotaxime, ceftazidime, ceftizoxime, ceftriaxone, and aztreonam were published in January 2010 (M100-S20) and are listed in Table 2A. Cefuroxime (parenteral) was also evaluated; however, no change in breakpoints was necessary with the dosage. When using the current breakpoints, routine ESBL testing is no longer necessary before reporting results (ie, it is no longer necessary to edit results for cephalosporins, aztreonam, or penicillins to resistant). However, ESBL testing may still be useful for epidemiological or infection control purposes. For laboratories that have not implemented the current breakpoints, ESBL testing should be performed as described in this table.

Note that breakpoints for drugs with limited availability in many countries (eg, moxalactam, cefonicid, cefamandole, and cefoperazone) were not evaluated. If considering use of these drugs for *E. coli, Klebsiella* spp., or *Proteus* spp., ESBL testing should be performed. If isolates test ESBL positive, the results for moxalactam, cefonicid, cefamandole, and cefoperazone should be reported as resistant.

Test	Criteria for Performance of ESBL Test		ESBL Test	
**Test method**	Disk diffusion	Broth microdilution	Disk diffusion	Broth microdilution
**Medium**	MHA	CAMHB	MHA	CAMHB
**Antimicrobial concentration**	For *K. pneumoniae, K. oxytoca,* and *E. coli*:<br>Cefpodoxime 10 µg or<br>Ceftazidime 30 µg or<br>Aztreonam 30 µg or<br>Cefotaxime 30 µg or<br>Ceftriaxone 30 µg<br><br>For *P. mirabilis*:<br>Cefpodoxime 10 µg or<br>Ceftazidime 30 µg or<br>Cefotaxime 30 µg<br><br>(Using more than one antimicrobial agent improves the sensitivity of ESBL detection.)	For *K. pneumoniae, K. oxytoca,* and *E. coli*:<br>Cefpodoxime 4 µg/mL or<br>Ceftazidime 1 µg/mL or<br>Aztreonam 1 µg/mL or<br>Cefotaxime 1 µg/mL or<br>Ceftriaxone 1 µg/mL<br><br>For *P. mirabilis*:<br>Cefpodoxime 1 µg/mL or<br>Ceftazidime 1 µg/mL or<br>Cefotaxime 1 µg/mL<br><br>(Using more than one antimicrobial agent improves the sensitivity of ESBL detection.)	Ceftazidime 30 µg<br>Ceftazidime-clavulanate[a] 30/10 µg<br><br><u>and</u><br><br>Cefotaxime 30 µg<br>Cefotaxime-clavulanate 30/10 µg<br><br>(Testing necessitates using both cefotaxime and ceftazidime, alone and in combination with clavulanate.)	Ceftazidime 0.25–128 µg/mL<br>Ceftazidime-clavulanate 0.25/4–128/4 µg/mL<br><br><u>and</u><br><br>Cefotaxime 0.25–64 µg/mL<br>Cefotaxime-clavulanate 0.25/4–64/4 µg/mL<br><br>(Testing necessitates using both cefotaxime and ceftazidime, alone and in combination with clavulanate.)
**Inoculum**	Standard disk diffusion procedure	Standard broth dilution procedure	Standard disk diffusion procedure	Standard broth dilution procedure
**Incubation conditions**	35°C ± 2°C; ambient air	35°C ± 2°C; ambient air	35°C ± 2°C; ambient air	35°C ± 2°C; ambient air
**Incubation length**	16–18 hours	16–20 hours	16–18 hours	16–20 hours

©Clinical and Laboratory Standards Institute. All rights reserved.

Table 3A. (Continued)

Test	Criteria for Performance of ESBL Test		ESBL Test	
**Test method**	Disk diffusion	Broth microdilution	Disk diffusion	Broth microdilution
**Results**	For *K. pneumoniae, K. oxytoca,* and *E. coli*:<br>Cefpodoxime zone ≤17 mm<br>Ceftazidime zone ≤22 mm<br>Aztreonam zone ≤27 mm<br>Cefotaxime zone ≤27 mm<br>Ceftriaxone zone ≤25 mm<br><br>For *P. mirabilis*:<br>Cefpodoxime zone ≤22 mm<br>Ceftazidime zone ≤22 mm<br>Cefotaxime zone ≤27 mm<br><br>Zones above may indicate ESBL production.	Growth at or above the concentrations listed may indicate ESBL production (ie, for *E. coli, K. pneumoniae,* and *K. oxytoca,* MIC ≥8 µg/mL for cefpodoxime or MIC ≥2 µg/mL for ceftazidime, aztreonam, cefotaxime, or ceftriaxone; and for *P. mirabilis,* MIC ≥2 µg/mL for cefpodoxime, ceftazidime, or cefotaxime).	A ≥5-mm increase in a zone diameter for either antimicrobial agent tested in combination with clavulanate vs the zone diameter of the agent when tested alone = ESBL (eg, ceftazidime zone = 16; ceftazidime-clavulanate zone = 21).	A ≥3 twofold concentration decrease in an MIC for either antimicrobial agent tested in combination with clavulanate vs the MIC of the agent when tested alone = ESBL (eg, ceftazidime MIC = 8 µg/mL; ceftazidime-clavulanate MIC = 1 µg/mL).
**Reporting**			For all confirmed ESBL-producing strains:<br><br>If laboratories do not use current cephalosporin and aztreonam breakpoints, the test interpretation should be reported as resistant for all penicillins, cephalosporins, and aztreonam.<br><br>If laboratories use current cephalosporin and aztreonam breakpoints, then test interpretations for these agents do not need to be changed from susceptible to resistant.	

©Clinical and Laboratory Standards Institute. All rights reserved.

Table 3A. (Continued)

Test	Criteria for Performance of ESBL Test		ESBL Test	
**Test method**	Disk diffusion	Broth microdilution	Disk diffusion	Broth microdilution
**QC recommendations**	When testing antimicrobial agents used for ESBL detection, *K. pneumoniae* ATCC®[b] 700603 is provided as a supplemental QC strain (eg, for training, competence assessment, or test evaluation). Either strain, *K. pneumoniae* ATCC® 700603 or *E. coli* ATCC® 25922, may then be used for routine QC (eg, weekly or daily).	When testing antimicrobial agents used for ESBL detection, *K. pneumoniae* ATCC® 700603 is provided as a supplemental QC strain (eg, for training, competence assessment, or test evaluation). Either strain, *K. pneumoniae* ATCC® 700603 or *E. coli* ATCC® 25922, may then be used for routine QC (eg, weekly or daily).	When performing the ESBL test, *K. pneumoniae* ATCC® 700603 and *E. coli* ATCC® 25922 should be used for routine QC (eg, weekly or daily).	When performing the ESBL test, *K. pneumoniae* ATCC® 700603 and *E. coli* ATCC® 25922 should be tested routinely (eg, weekly or daily).
	E. coli ATCC® 25922 (see acceptable QC ranges in Table 4A-**1**)	*E. coli* ATCC® 25922 = No growth (see acceptable QC ranges listed in Table 5A-**1**)	**Acceptable QC:**<br>*E. coli* ATCC® 25922: ≤ 2-mm increase in zone diameter for antimicrobial agent tested in combination with clavulanate vs the zone diameter when tested alone.	**Acceptable QC:**<br>*E. coli* ATCC® 25922: < 3 twofold concentration decrease in MIC for antimicrobial agent tested in combination with clavulanate vs the MIC of the agent when tested alone.
	K. pneumoniae ATCC® 700603:<br>Cefpodoxime zone 9–16 mm<br>Ceftazidime zone 10–18 mm<br>Aztreonam zone 9–17 mm<br>Cefotaxime zone 17–25 mm<br>Ceftriaxone zone 16–24 mm	*K. pneumoniae* ATCC® 700603 = Growth:<br>Cefpodoxime MIC ≥ 8 µg/mL<br>Ceftazidime MIC ≥ 2 µg/mL<br>Aztreonam MIC ≥ 2 µg/mL<br>Cefotaxime MIC ≥ 2 µg/mL<br>Ceftriaxone MIC ≥ 2 µg/mL	*K. pneumoniae* ATCC® 700603:<br>≥ 5-mm increase in zone diameter of ceftazidime-clavulanate vs ceftazidime alone; ≥ 3-mm increase in zone diameter of cefotaxime-clavulanate vs cefotaxime alone.	*K. pneumoniae* ATCC® 700603:<br>≥ 3 twofold concentration decrease in MIC for an antimicrobial agent tested in combination with clavulanate vs the MIC of the agent when tested alone.

Abbreviations: ATCC®, American Type Culture Collection; CAMHB, cation-adjusted Mueller-Hinton broth; ESBL, extended-spectrum β-lactamase; MHA, Mueller-Hinton agar; MIC, minimal inhibitory concentration; PK-PD, pharmacokinetic-pharmacodynamic; QC, quality control.

Footnotes

a. Preparation of ceftazidime-clavulanate (30 µg/10 µg) and cefotaxime-clavulanate (30 µg/10 µg) disks: Using a stock solution of clavulanate at 1000 µg/mL (either freshly prepared or taken from small aliquots that have been frozen at −70°C), add 10 µL of clavulanate to ceftazidime (30 µg) and cefotaxime (30 µg) disks. Use a micropipette to apply the 10 µL of stock solution to the ceftazidime and cefotaxime disks within one hour before they are applied to the plates, allowing about 30 minutes for the clavulanate to absorb and the disks to be dry enough for application. Use disks immediately after preparation or discard; do not store.

b. ATCC® is a registered trademark of the American Type Culture Collection.

©Clinical and Laboratory Standards Institute. All rights reserved.
